# Motor neurons and endothelial cells additively promote development and fusion of human iPSC-derived skeletal myocytes

**DOI:** 10.1186/s13395-024-00336-4

**Published:** 2024-03-07

**Authors:** Suradip Das, Melanie C. Hilman, Feikun Yang, Foteini Mourkioti, Wenli Yang, D. Kacy Cullen

**Affiliations:** 1 Department of Neurosurgery, Center for Brain Injury & Repair, Perelman School of Medicine, University of Pennsylvania, Philadelphia, PA 19104 USA; 2 Center for Neurotrauma, Neurodegeneration & Restoration, Corporal Michael J. Crescenz Veterans Affairs Medical Center, Philadelphia, PA 19104 USA; 3 Department of Bioengineering, School of Engineering and Applied Science, University of Pennsylvania, Philadelphia, PA USA; 4 Department of Medicine, Penn Institute for Regenerative Medicine, Cardiovascular Institute, Perelman School of Medicine, University of Pennsylvania, Philadelphia, PA USA; 5 Department of Orthopaedic Surgery, Perelman School of Medicine, University of Pennsylvania, Philadelphia, PA USA; 6 Department of Cell and Developmental Biology, Perelman School of Medicine, University of Pennsylvania, Philadelphia, PA USA; 7 Musculoskeletal Program, Penn Institute for Regenerative Medicine, Perelman School of Medicine, University of Pennsylvania, Philadelphia, PA 19104 USA

## Abstract

**Background:**

Neurovascular cells have wide-ranging implications on skeletal muscle biology regulating myogenesis, maturation, and regeneration. Although several in vitro studies have investigated how motor neurons and endothelial cells interact with skeletal myocytes independently, there is limited knowledge about the combined effect of neural and vascular cells on muscle maturation and development.

**Methods:**

Here, we report a triculture system comprising human-induced pluripotent stem cell (iPSC)-derived skeletal myocytes, human iPSC-derived motor neurons, and primary human endothelial cells maintained under controlled media conditions. Briefly, iPSCs were differentiated to generate skeletal muscle progenitor cells (SMPCs). These SMPCs were seeded at a density of 5 × 10^4^ cells/well in 12-well plates and allowed to differentiate for 7 days before adding iPSC-derived motor neurons at a concentration of 0.5 × 10^4^ cells/well. The neuromuscular coculture was maintained for another 7 days in coculture media before addition of primary human umbilical vein endothelial cells (HUVEC) also at 0.5 × 10^4^ cells/well. The triculture was maintained for another 7 days in triculture media comprising equal portions of muscle differentiation media, coculture media, and vascular media. Extensive morphological, genetic, and molecular characterization was performed to understand the combined and individual effects of neural and vascular cells on skeletal muscle maturation.

**Results:**

We observed that motor neurons independently promoted myofiber fusion, upregulated neuromuscular junction genes, and maintained a molecular niche supportive of muscle maturation. Endothelial cells independently did not support myofiber fusion and downregulated expression of *LRP4* but did promote expression of type II specific myosin isoforms. However, neurovascular cells in combination exhibited additive increases in myofiber fusion and length, enhanced production of Agrin, along with upregulation of several key genes like *MUSK*, *RAPSYN*, *DOK-7*, and *SLC2A4*. Interestingly, more divergent effects were observed in expression of genes like *MYH8*, *MYH1*, *MYH2*, *MYH4*, and *LRP4* and secretion of key molecular factors like amphiregulin and IGFBP-4.

**Conclusions:**

Neurovascular cells when cultured in combination with skeletal myocytes promoted myocyte fusion with concomitant increase in expression of various neuromuscular genes. This triculture system may be used to gain a deeper understanding of the effects of the neurovascular niche on skeletal muscle biology and pathophysiology.

**Supplementary Information:**

The online version contains supplementary material available at 10.1186/s13395-024-00336-4.

## Background

Skeletal muscle tissue is irrigated with a dense array of microvasculature and an axonal network to sustain its high metabolic demands and enable its contractile function, respectively. The microvascular unit within myofibers comprises of endomysial capillaries running parallel to myofibers. These capillaries are fed by one terminal arteriole and drained by one venule and represents the smallest functional unit regulating blood flow within a skeletal muscle [1]. Further, most extrafusal muscle fibers are connected via neuromuscular junctions to a single alpha motor neuron arising from the spinal cord, thereby forming the motor unit which is the smallest force-generating unit of the skeletal muscle [2]. The microvascular unit and the motor unit together ensure continuous perfusion and enable function of skeletal tissue. However, vascularization and innervation have been reported to have a much broader impact on skeletal muscle biology beyond nutrient supply and function by each being suggested to influence myogenesis, maturation, and regeneration.

Both the myogenic and endothelial cells trace their embryonic origin to the dermomyotome which is an epithelial-like structure giving rise to the dermis, muscle fibers, and endothelial cells. A rudimentary dense network of capillaries forms during prenatal development in muscles regulated by the Notch signaling pathway. During postnatal development, this mesh of short, interconnected capillaries reorganizes and forms a mature microvascular network running parallel to myofibers. Myogenic and endothelial cells are privileged partners and reciprocally interact to promote myogenesis and angiogenesis [3]. During muscle regeneration, vascular cells like endothelial cells and pericytes interact with resident stem cell population, i.e., satellite cells to regulate myogenesis. Specifically, peri-endothelial cells like smooth muscle cells and pericytes surround the endothelial cells and keep the muscle satellite cells in quiescence [4]. Muscle injury and consequent damage to the microvasculature free up the endothelial cells to interact directly with myogenic cells and promote myogenesis and angiogenesis [5]. The pro-myogenic role of endothelial cells has been utilized in developing pre-vascularized tissue-engineered muscle grafts which promoted satellite cell migration within the injury site and augmented tissue repair [6, 7].

Neural input not only controls muscle function, but appropriate and timely innervation is critical for development, maturation, and regeneration of skeletal muscles [8]. During prenatal development, acetylcholine receptors are dispersed along the surface of myofibers. As a myofiber receives motor axon(s), the acetylcholine receptors that synapse with axon terminals stabilize, enlarge, and form neuromuscular junctions (NMJs), whereas extra-junctional receptors disperse over time [9, 10]. By the end of prenatal period, myofibers are poly-innervated being connected to multiple motor neurons. During postnatal development, neurons are pruned such that one myofiber has a single synaptic contact with an axon terminal. Subsequently, the innervated acetylcholine receptors undergo morphological transformation from oval-shaped plaque morphology to a more matured pretzel-shaped structure [7, 9]. Most muscle injury and diseases are associated with local axotomy leading to chronic denervation which limits functional regeneration. Our group has previously developed innervated tissue-engineered muscle comprised of a preformed network of motor axons for implantation in a rodent model of severe musculoskeletal trauma like volumetric loss [11]. This work demonstrated how the presence of motor neurons promotes myocyte fusion in vitro and facilitates migration of host satellite cells and endothelial cells within the injury area, thereby maintaining a pro-regenerative milieu following muscle trauma [11].

Skeletal myocytes have been cultured with neural and vascular cells separately to understand the effect of innervation and vascularization independently on myocyte development and function. Cocultures of adult skeletal myocytes and endothelial cells have been reported with or without the addition of fibroblasts primarily for fabrication of pre-vascularized tissue-engineered muscle. Most of these studies focused on optimizing media conditions for vascular network formation in vitro which can subsequently promote in vivo angiogenesis and muscle regeneration [6, 7]. However, the effect of endothelial cells on skeletal myocyte development and myofiber maturation is still not fully understood. The effect of innervation on skeletal myocytes is well-established with numerous studies utilizing neuromuscular cocultures both in monolayer or three-dimensional (3D) constructs as models for understanding the biology of NMJs and neuron-mediated control of muscle contraction [12–16].

While neural and vascular implications on skeletal muscles have been studied separately, there is a need to develop an in vitro triculture system that facilitates understanding of interactions between these three tissue types. In this article, we report a novel myoneurovascular triculture system comprising human iPSC-derived myocytes and motor neurons along with primary human endothelial cells to investigate the effect of innervation and vascularization on skeletal myocyte maturation as well as how myocytes impact growth of neural and vascular cells. This system is further utilized to perform gene expression analysis to understand neurovascular implications on myofiber type as well as expression of NMJ-related genes.

## Methods

Human iPSC line (Penn123i-SV20) [17] available at the University of Pennsylvania iPSC Core was used to derive skeletal myocyte progenitors. Human iPSC-derived spinal motor neurons were purchased from BrainXell (BX-0100), and human umbilical vein endothelial cells (HUVEC) was purchased from ATCC (PCS-100–010).

### Generation of human iPSC-derived skeletal myotubes

Myogenic differentiation of hiPSCs to skeletal muscle cells was performed by adapting two previously published protocols [18, 19]. We followed the differentiation protocol outlined by Chal et al. to generate Pax-7 + skeletal muscle progenitor cells (SMPCs) within 30 days [19]. This was followed by enrichment of myogenic progenitors based on expression of ERBB3/NGFR that has been previously reported by Hicks et al. to demarcate myogenic populations [18].

Briefly, single hiPSCs were seeded into 6-well plates at a density of 3 × 10^5^ cells per well in StemMACS iPS-Brew medium (Miltenyi Biotec, 130–104-368) for 24 h before the initiation of differentiation. At differentiation days between 28 and 32, HNK1^−^ERBB3^+^NGFR^+^ cells were collected and cultured in skeletal muscle growth media (see Table 1) with medium change every other day. When the SMPCs reached 70–80% confluency (designated as passage 0), they were expanded and then cryopreserved.

**Table 1:** Details of different cell culture media used

Media type	Media composition
Skeletal muscle growth media	SKGM2 medium (Lonza) + bFGF (10 ng/mL, Thermo Fisher, PHG0023)
Skeletal muscle differentiation media	DMEM/F12 (Thermo Fisher, 11,330), ITS-G (Thermo Fisher, 41,400,045), N2 supplement (Thermo Fisher, 17,502,048), Pen/Strep, and L-glutamine supplemented with 10-µM TGF-b inhibitor SB-431542 (Tocris, 1614) and 10 ng/ml IGF-1 (R&D Systems, 291-G1)
Coculture media or motor neuron media	Neurobasal + 37‐ng/ml hydrocortisone, 2.2‐μg/ml isobutylmethylxanthine, 10‐ng/ml brain‐derived neurotrophic factor, 10‐ng/ml ciliary neurotrophic factor, 10‐ng/ml CT‐1, 10‐ng/ml glial cell‐derived neurotrophic factor, 2% B‐27, 20‐ng/ml nerve growth factor, 20‐μM mitotic inhibitors, 2‐mM L‐glutamine, 417‐ng/ml forskolin, 1‐mM sodium pyruvate, 0.1‐mM β‐mercaptoethanol, and 2.5‐g/L glucose
Vascular media	Vascular cell basal medium (ATCC PCS-100–030) + endothelial growth kit-VEGF (ATCC PCS-100–041) comprising of the following: rh VEGF: 5 ng/mL, rh EGF: 5 ng/mL, rh FGF basic: 5 ng/mL, rh IGF-1: 15 ng/mL, L-glutamine: 10 mM, heparin sulfate: 0.75 units/mL, hydrocortisone: 1 µg/mL, ascorbic acid: 50 µg/mL, fetal bovine serum: 2%
Triculture media	Skeletal muscle differentiation tool (ATCC PCS-950–050) + coculture media + vascular media = 1:1:1

### Culture of myocytes, motor neurons, and endothelial cells

To generate myotubes, cell culture surface was coated with 20 µg/mL poly-D-lysine (PDL) in sterile cell culture water overnight. The surface was subsequently washed thrice with PBS before coating with laminin (20 µg/mL) for 2 h. SMPCs between passages 3 and 6 were seeded at a density of 5 × 10^4^ cells/well in 12-well plates in SKGM2 medium supplemented with bFGF for 2 days and then differentiated in differentiation medium for additional 5 days until 7 days in vitro (DIV) to generate skeletal myotubes (MYO) (see Table 1). Human iPSC-derived motor neurons (MN, purchased from BrainXell) were plated on top of the myocyte layer at a concentration of 0.5 × 10^4^cells/well, and the coculture was maintained with coculture media (Table 1) up to 14 DIV with regular changes of media. Subsequently, HUVEC (EC) was added to the neuromuscular coculture at a concentration of 0.5 × 10^4^ cells/well, and the triculture (*MYO* + *MN* + *EC*) was maintained for another 7 days until 21 DIV in triculture media (Table 1). The cell seeding densities and total culture duration were kept same for all the experimental groups to maintain parity of culture condition. Specifically, for the *MYO* + *MN* group, myoblasts were allowed to differentiate skeletal muscle differentiation media for 7 DIV as described earlier prior to addition of human iPSC motor neurons. The neuromuscular culture was maintained in coculture medium for 7 days (14 DIV) followed by maintenance in triculture media until 21 DIV. Similarly, for the *MYO* + *EC* group, myoblasts were differentiated for 7 days in differentiation media followed by another 7-day culture in coculture media (for maintaining parity between groups) and finally addition of HUVEC cells and myovascular coculture in triculture media until 21 DIV.

### Immunofluorescent staining of cells

Samples were fixed for 35 min in 4% paraformaldehyde (EMS, cat. no. 15710), washed three times with 1 × PBS, and permeabilized in 0.3% Triton-X100 + 4% normal horse serum (NHS) (Sigma) for 60 min. Samples were blocked in 4% NHS (Sigma), and all subsequent steps were performed using 4% NHS for antibody dilutions. For staining of actin and acetylcholine receptors, samples were incubated with AlexFluor-488-conjugated phalloidin (1:200, Invitrogen, A12379) and AlexaFluor-647-conjugated bungarotoxin (1:250, Invitrogen, B35450) respectively. For morphological assessment of motor neurons and endothelial cells, fixed samples were incubated with an axonal marker SMI-35 (1:250, Abcam, ab18207) or motor neuron-specific marker for choline acetyltransferase (ChAT) (1:200, Abcam, ab18736), presynaptic marker synaptophysin (1:500, Abcam, ab32127), and endothelial surface marker CD31 (1:500, Abcam, ab32127) for 16 h at 4 °C followed by AlexaFluor-568 antibody and AlexaFluor-647 antibody (Life Technologies). Images were acquired using a Nikon Eclipse TI A1RSI laser scanning confocal microscope.

### Quantification of myocyte fusion index, myocyte length, and myocyte width

MYO (*n* = 3), MYO + MN (*n* = 3), MYO + MN + EC (*n* = 3), and MYO + EC (*n* = 3) cultures were considered for measuring fiber length, fiber width, and myocyte fusion index (MFI) as per the following equation:

$$\text{MFI}=\frac{\mathrm{Number}\;\mathrm{of}\;\mathrm{nuclei}\;\mathrm{in}\;\mathrm{myocytes}\;\mathrm{with}\;\mathrm{more}\;\mathrm{than}\;\mathrm{three}\;\mathrm{nuclei}}{\mathrm{Total}\;\mathrm{number}\;\mathrm{of}\;\mathrm{nuclei}\;\mathrm{within}\;\mathrm{myocytes}}$$

Myocytes within 500 μm × 500 μm in each sample were considered for calculating MFI, fiber length, and fiber width, and the mean was plotted for each group (Fig. 2C).

### Quantification of myofiber alignment

MYO (*n* = 3), MYO + MN (*n* = 3), MYO + MN + EC (*n* = 3), and MYO + EC (*n* = 3) cultures were utilized to measure myofiber alignment. The phalloidin channel (for myocytes) was included in the analysis. The images (1.5 mm × 1.5 mm ROIs) were converted to grayscale in FIJI and then processed using the Directionality plugin to measure the angle of orientation. A fast Fourier transform method was used across a starting angle of − 90° and end angle + 90° with number of bins = 90. The raw data was imported in GraphPad Prism to plot histograms and perform a nonlinear fit curve analysis (Gaussian).

### Gene expression analysis

All the genetic analyses were performed simultaneously from the same batch of cells. Total RNA was isolated after 21 days of culture using a TRIzol reagent (Fisher Scientific, Hampton, NH, USA) according to the manufacturer’s instructions. One microgram of RNA was used for complementary DNA (cDNA) synthesis by using High-Capacity cDNA Reverse Transcription Kit (Thermo Fisher Scientific). qPCR was carried out with 5 ng of cDNA per reaction using SYBR Green PCR Master Mix (Fisher Scientific, USA) on QuantStudio™ 6 Flex real time PCR system. Human TATA-binding protein (hTBP) was used as reference gene to normalize gene expression levels. The PCR primers are listed in Table 2.

**Table 2:** Details of primers for gene expression analysis

**Group**	**Gene**	Primer (forward)	Primer (reverse)
Myosin heavy chain isoforms	*MYH1*	AATGTCCAAGGCCAACAGTGA	AGCATCCTGCAGACGCTGA
	*MYH2*	GGAGGACAAAGTCAACACCCTG	GCCCTTTCTAGGTCCATGCGAA
	*MYH3*	CTGGAGGATGAATGCTCAGAGC	CCCAGAGAGTTCCTCAGTAAGG
	*MYH4*	GACAGCCAAGAAGAGGAAACTGG	ACCTGCCATCTCTTCTGTGAGG
	*MYH6*	GGAAGACAAGGTCAACAGCCTG	TCCAGTTTCCGCTTTGCTCGCT
	*MYH7*	GGAGTTCACACGCCTCAAAGAG	TCCTCAGCATCTGCCAGGTTGT
	*MYH8*	GGAGCAAGCTGAGCCAGATG	CACAGTCTGGCCTTTGGTGA
Developmental	*Pax7*	GGAGGATGAAGCGGACAAGAAG	AGGTCAGGTTCCGACTCCACAT
	*MYOD1*	CTCCAACTGCTCCGACGGCAT	ACAGGCAGTCTAGGCTCGACAC
	*MYOG*	AGTGCCATCCAGTACATCGAGC	AGGCGCTGTGAGAGCTGCATTC
	*DMD*	GCTCAACCATCGATTTGCAGCC	TTCAGCCTCCAGTGGTTCAAGC
Structural	*TNNT1*	AACGCGAACGTCAGGCTAAGCT	CTTGACCAGGTAGCCGCCAAAA
	*TTN*	CTGCTGACTACACCTTTGTGGC	GCTCGCTTCTTCTCCAGTACCT
Glucose transporter	*SLC2A1*	TTGCAGGCTTCTCCAACTGGAC	CAGAACCAGGAGCACAGTGAAG
	*SLC2A4*	CCATCCTGATGACTGTGGCTCT	GCCACGATGAACCAAGGAATGG
Neuromuscular	*MUSK*	TTTGCTGTCCGTGCCAGAATGC	GGCTTTGAGGACGTCATTGGTG
	*LRP4*	GTGTGGCAGAACCTTGACAGTC	ACCGCTCTAACTTGGCATTCTCC
	*RAPSYN*	GGACAAAGGTGCTGGAGAAGAG	TGTCGATCTGGACCACAGCGAA
	*DOK7*	GCCATCATGCTGGGCTTTGACA	AACTTGGTGCCTGGAGCCACTG

### Biomolecule analysis

#### Quantibody-based assay

Cell culture supernatants were collected from each sample at 21 DIV. Regular triculture media was used as control group. At least three replicates from each group were considered for analysis. The supernatants were passed through 0.22-μm filters to remove any cellular debris, frozen, and shipped to RayBiotech facility for Quantibody-based Human Growth factor Assay (QAH-GF-1–2). The final level of growth factors was obtained by subtracting the control triculture media levels from the values of the experimental samples. Although the full quantibody assay can measure around 40 growth factors, only those that were above detection limit after subtracting background media levels are reported here.

#### Agrin ELISA assay

Cell culture supernatants were collected from each sample at 21 DIV (*n* = 6/group) and processed as mentioned earlier. Regular triculture media was used as control group. Human Agrin ELISA kit (Abcam, ab216945) was used to quantify Agrin levels following manufacturer’s protocol.

### Statistical analysis

All quantifications reported in this study were performed by personnel blinded about the treatment groups. All statistical analysis was performed using GraphPad Prism software. For comparison between multiple groups, a one-way analysis of variance (ANOVA) or Kruskal-Wallis test was performed with post hoc Tukey’s adjustment (Figs. 2C, 5A, B, 6A and 7B) or Dunn’ multiple comparison test (7A). Significance was taken at *p* ≤ 0.05 (*), *p* ≤ 0.01 (**), *p* ≤ 0.001 (***), and *p* ≤ 0.0001 (****). All graphs were made in GraphPad Prism and display mean ± standard error of mean (SEM).

## Results

### ERBB3^+^/NGFR^+^ population of human iPSC-derived myoblasts exhibits rapid differentiation to myotubes

**Fig. 1:**
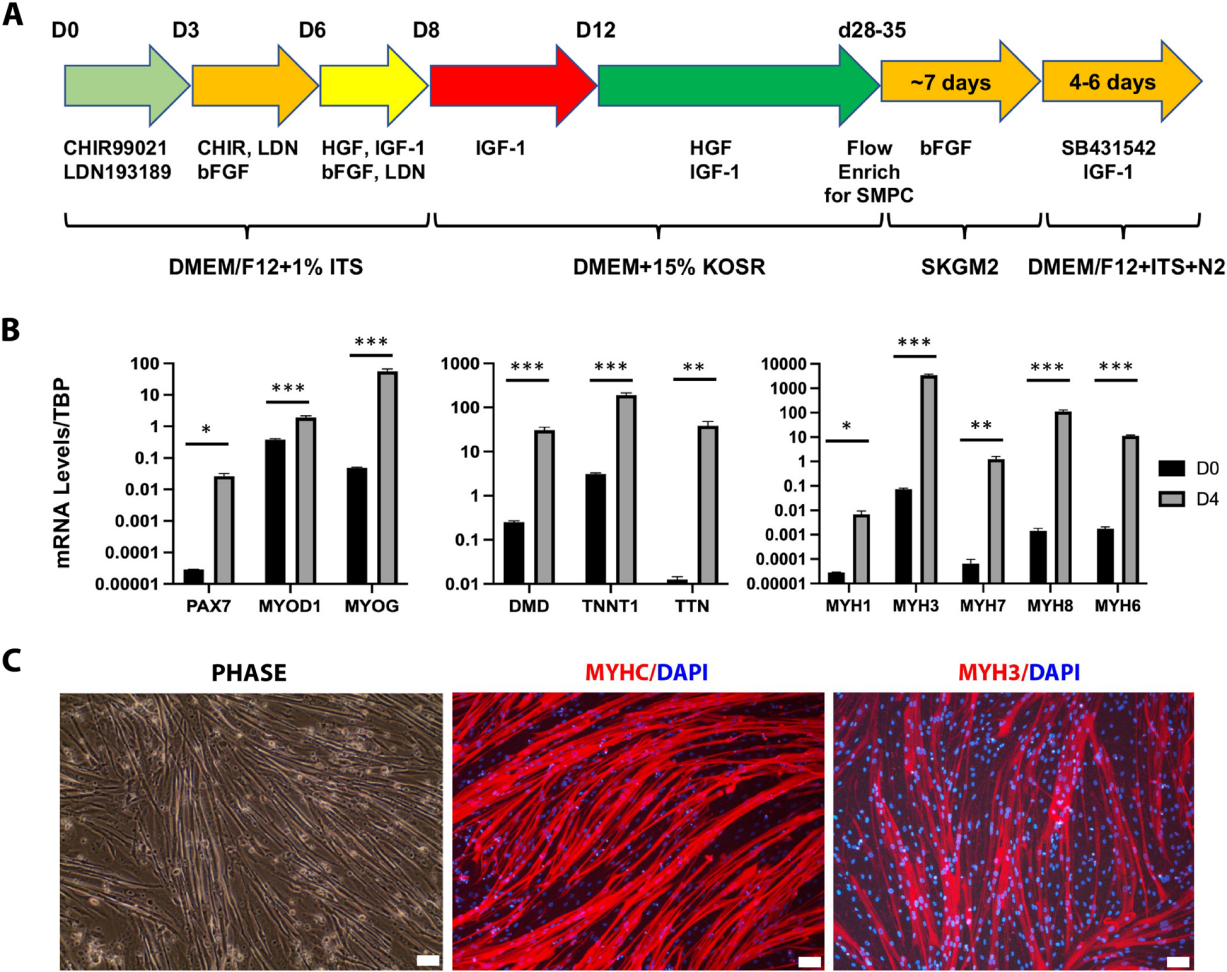
Generation of human iPSC-myotubes. **A** Schematic of differentiation of human iPSC myotubes using protocol described in Chal et al. [19] and Hicks et al. [18]. Skeletal muscle progenitor cells (SMPCs) are differentiated from iPSCs and enriched by flow sorting. The SMPCs are then expanded in SKGM2 media and differentiated to form myotubes. **B** Gene expression analysis by qRT-PCR of undifferentiated SMPCs (D0) and day 4 differentiated myotubes (D4). **C** Phase-contrast picture and immunocytochemistry staining of day 4 differentiated iPSC myotubes. MYHC, pan myosin heavy chain; MYH3, myosin heavy chain 3. Scale bar — 10 μm (phase) and 20 μm (fluorescence)

Human-induced pluripotent stem cell line Penn123i-SV20 [17] available at Penn iPSC Core was differentiated into a heterogenous population of myocytes, myotube, and skeletal muscle progenitor cells (SMPCs) following previously published protocol [18, 19]. After enriching for cells using antibodies against cell surface markers ERBB3 and NGFR, which mark PAX7^+^ myogenic progenitor cells, we expanded and cryopreserved the skeletal muscle progenitor cells (SMPCs) for all subsequent fusion into myotubes. The ERBB3^+^NGFR^+^ cells were able to convert to myotubes with high efficiency within 4 days upon initiation of secondary differentiation to myotubes (Fig. 1).

Quantitative PCR analysis revealed significant increases of myogenic-related transcription factor genes (*PAX7*, *MYOD1*, and *MYOG*), structural protein genes (*DMD*, *TNNT1*, *TTN*), and various muscle fiber subtype genes (*MYH1*, *MYH3*, *MYH6*, *MYH7*, and *MYH8*) (Fig. 1B) consistent with previous report [18]. Myogenic phenotype of cell population was further confirmed by uniform staining of the skeletal muscle myosin heavy chain (MYHC) and the embryonic myosin heavy chain MYH3 (Fig. 1C). Based on these results, we used an ERBB3^+^/NGFR^+^ enriched myoblast population as our starting cell source for all future experiments.

### Neurovascular cells additively promote myocyte fusion and elongation

**Fig. 2:**
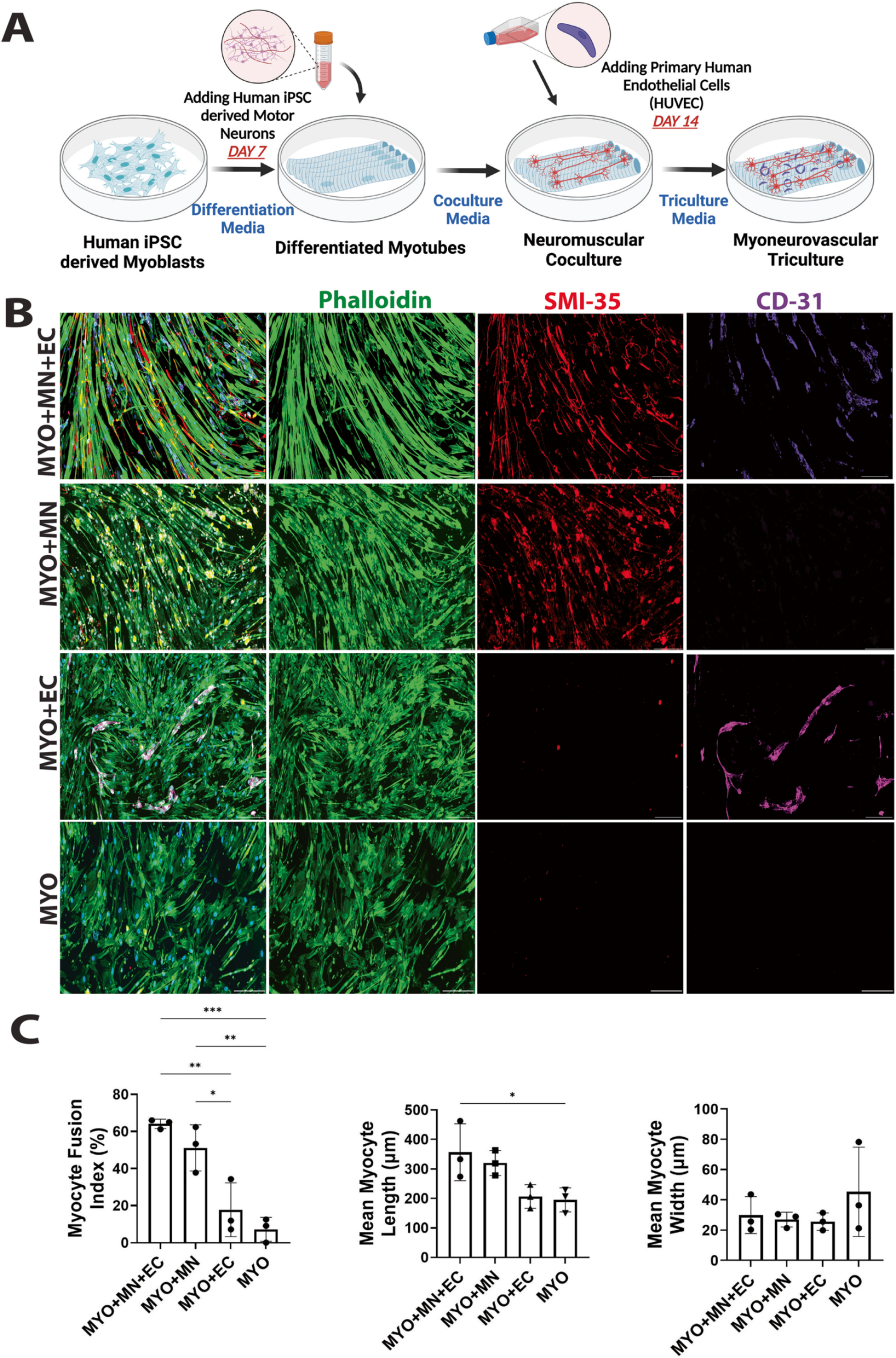
Development and morphological characterization of myoneurovascular tri-culture. **A** Schematic demonstrating our cell culture approach. Myoneurovascular triculture was generated by differentiating human iPSC-derived myocytes for 7 days in differentiation media to allow formation of myotubes before adding human iPSC-derived motor neurons to the culture. The combined neuromuscular culture was maintained for a further 7 days in coculture media before adding HUVEC endothelial cells. Finally, the three cell types were cultured for another 7 days in triculture media. **B** Human iPSC-derived myocytes (MYO), motor neurons (MN), and primary endothelial cells (EC) were cultured in various combinations under optimized conditions for 21 DIV and phenotypically characterized using cell-specific markers. Myocytes were stained for F-actin using phalloidin, motor neurons were stained for expression of hypo-phosphorylated neurofilament H using SMI-35 antibody, and CD31 was used as marker for endothelial cells. Neurons and endothelial cells did not exhibit any significant morphological change when grown individually (MYO + MN/MYO + EC) or together (MYO + MN + EC) on a bed of pre-differentiated myocytes. However, the iPSC myocytes demonstrated a more elongated and aligned architecture when cultured in the presence of motor neurons (MYO + MN and MYO + MN + EC), as compared to when grown only in the presence of ECs (MYO + EC) or as a monoculture (MYO). Scale bar — 250 µm. **C** Morphological parameters of myocytes were monitored to investigate the neurovascular implications on myocyte development. Cellular fusion leading to polynucleated myofibers was analyzed by measuring myocyte fusion index. Motor neurons significantly increased MFI (MYO + MN) over myocyte monocultures as compared to endothelial cell-myocyte coculture group (MYO + EC). Of note, addition of endothelial cells on neuromuscular coculture group (MYO + MN + EC) further enhanced myocyte fusion, but this effect was not significant in comparison to fusion index observed in neuromuscular cocultures (MYO + MN). Further, simultaneous coculture of motor neurons and endothelial cells contributed to elongation of myofibers but had no effect on their thickness. *n* = 3/group, *p* < 0.05 for significance

**Fig. 3:**
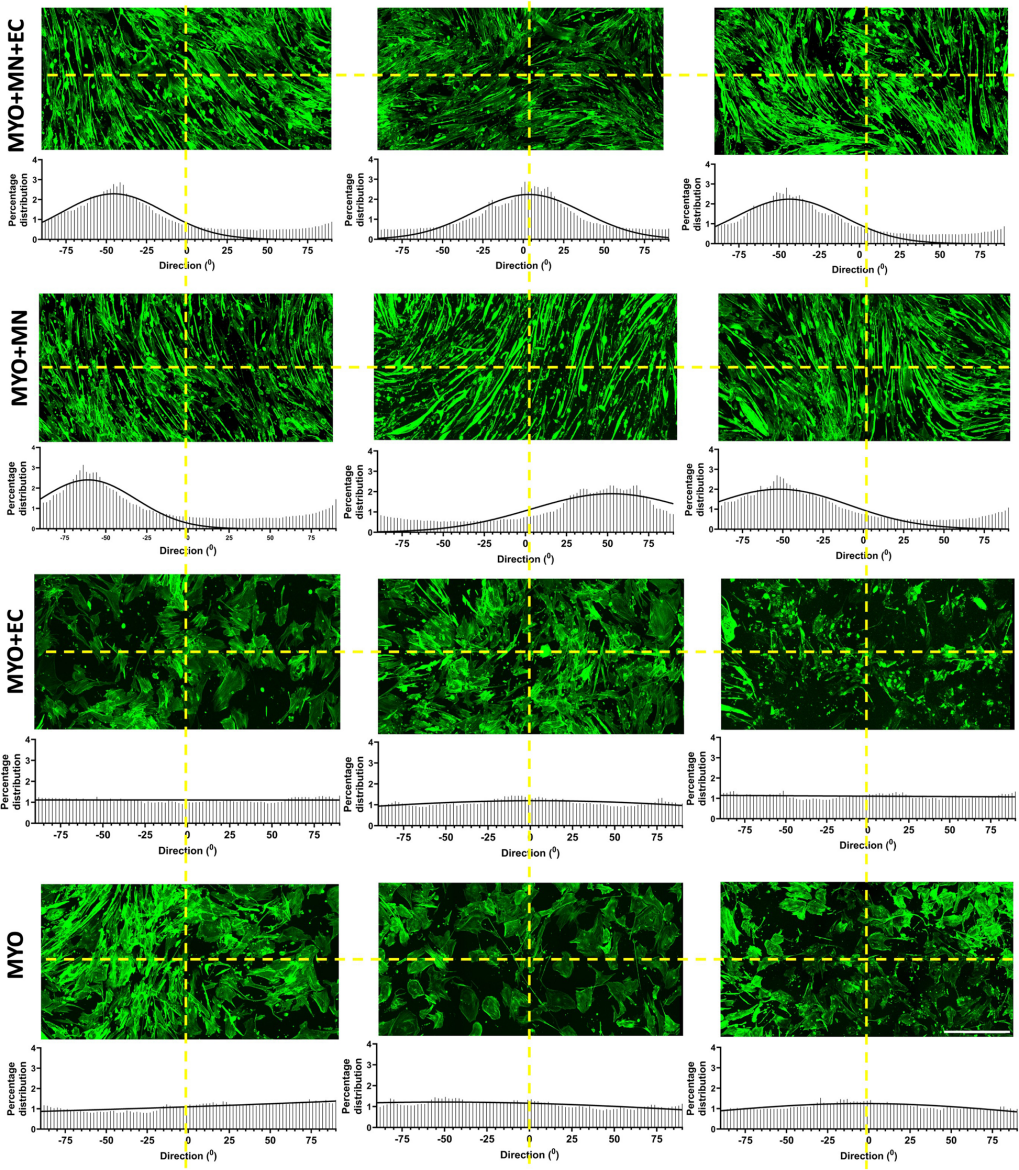
Motor neurons promote alignment of skeletal myocytes. The orientation of skeletal myocytes across all the groups — MYO + MN + EC, MYO + MN, MYO + EC, and MYO, was measured using directionality plug-in in ImageJ. The angle of orientation from three replicates per group was plotted as histograms. Cultures with motor neurons (i.e., MYO + MN and MYO + MN + EC) promoted the formation of anisotropic clusters of myofibers. The yellow broken lines indicate the reference orientation of the central 0-degree angle (horizontal line and the vertical are 90°). Scale bar — 500 μm

Human iPSC-derived SMPCs were allowed to differentiate for 7 days (MYO) before seeding iPSC-derived motor neurons (MN). The neuromuscular coculture was maintained for another 7 days before adding human umbilical vein-derived endothelial cells (HUVECs), and the triculture was terminated after another 7 days. The myocytes, motor neurons, and endothelial cells were observed to express characteristic phenotypic markers like F-actin, hypo-phosphorylated neurofilament, and CD31 respectively across all combination groups (Fig. 2A). This demonstrates that the timing and densities of cell seeding along with recipe of triculture media comprising equal proportions of muscle differentiation media, motor neuron media, and vascular media (Table 1) were suitable for growth and survival of myoneurovascular cells. Neurons and endothelial cells did not exhibit any significant morphological change when grown separately with myocytes (MYO + MN or MYO + EC) (Fig. 2A). Motor neurons were found to promote alignment of myofibers in the MYO + MN and MYO + MN + EC groups (Fig. 3). Morphological parameters like length and width of myofibers as well as proportion of polynucleated myofibers were quantified to understand neurovascular implications on maturation of myofibers. Motor neurons alone as well as in combination with endothelial cells increased fusion, length, and alignment of myofibers (MYO + MN, MYO + MN + EC) (Fig. 2B, Fig. 3). Interestingly, addition of endothelial cells alone did not significantly improve fusion or increase elongation of myocytes in MYO + EC cultures. Neither neural nor vascular cells affected myofiber diameter (Fig. 2B). These results indicate that motor neurons and endothelial cells in combination facilitate morphological maturation of myofibers.

### Myocyte conditioning of triculture media facilitates survival of neurovascular cells

**Fig. 4:**
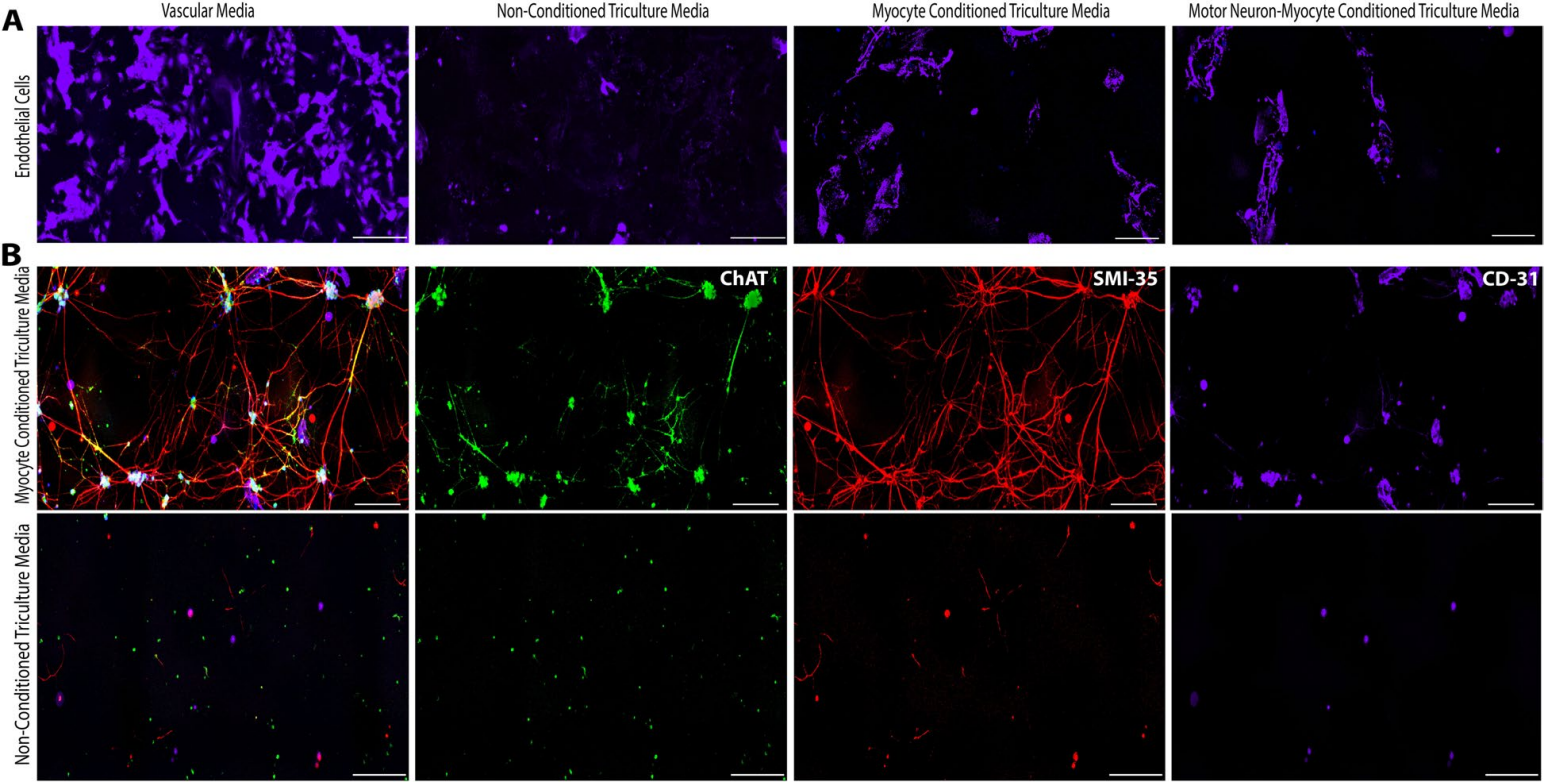
Effect of myocyte-conditioned media on neural and endothelial cell cultures. **A**)Endothelial cells (HUVEC) were cultured as a monolayer in various media formulations to study the effect of myocyte conditioned or motor neuron-myocyte coculture conditioned media on endothelial cell survival and growth through expression of CD31 marker (purple). Nonconditioned triculture media was found to be not conducive for endothelial cell growth. Media conditioned with either myocytes only or myocyte-motor neuron coculture was observed to promote endothelial cell survival and growth like the recommended media for HUVEC (vascular media) used here as a positive control. Scale bar — 250 µm. **B** Myocyte-conditioned triculture media was used to maintain a coculture of motor neurons and endothelial cells for 14 div to study the effect of myocyte conditioning on neurovascular health. Motor neurons and endothelial cells improved cell morphology and growth as evidenced by expression of mature motor neuron markers (ChAT and SMI-35) and endothelial marker (CD31), when cultured in myocyte-conditioned triculture media. Nonconditioned triculture media was found to be not supportive of neuronal or endothelial cell growth. Scale bar — 250 µm

Endothelial cells (HUVECs) were cultured on PDL/laminin-coated multiwell plates for 7 days under different media combinations. Nonconditioned base triculture media appeared to be not supportive for endothelial cell cultures. Conditioning the triculture media for 24 h with only myocytes or myocyte-motor neuron coculture appeared to significantly improve endothelial cell attachment and proliferation with evidence of tube-like structures (Fig. 4A). Separately, a coculture of motor neurons and endothelial cells were generated by initially plating motor neurons on PDL/laminin-coated multiwell plates for 7 days followed by addition of endothelial cells and maintaining the coculture for another 7 days in either conditioned or nonconditioned triculture media. We found that like the endothelial cell monoculture, the nonconditioned triculture media formulation did not support growth of motor neurons. However, 24-h conditioning of the triculture media with myocytes drastically improved motor neuron attachment, neurite outgrowth, and endothelial cell growth (Fig. 4B). Altogether, the results demonstrate that conditioning our triculture media formulation with myocytes greatly facilitates the growth of motor neurons and endothelial cells, further supporting the rationale that seeding myocytes first followed by motor neurons and endothelial cells is an appropriate system for generating the myoneurovascular tricultures.

### Motor neurons and endothelial cells show differential effects on expression of genes related to muscle fiber type, muscle structure, and glucose transporters

**Fig. 5:**
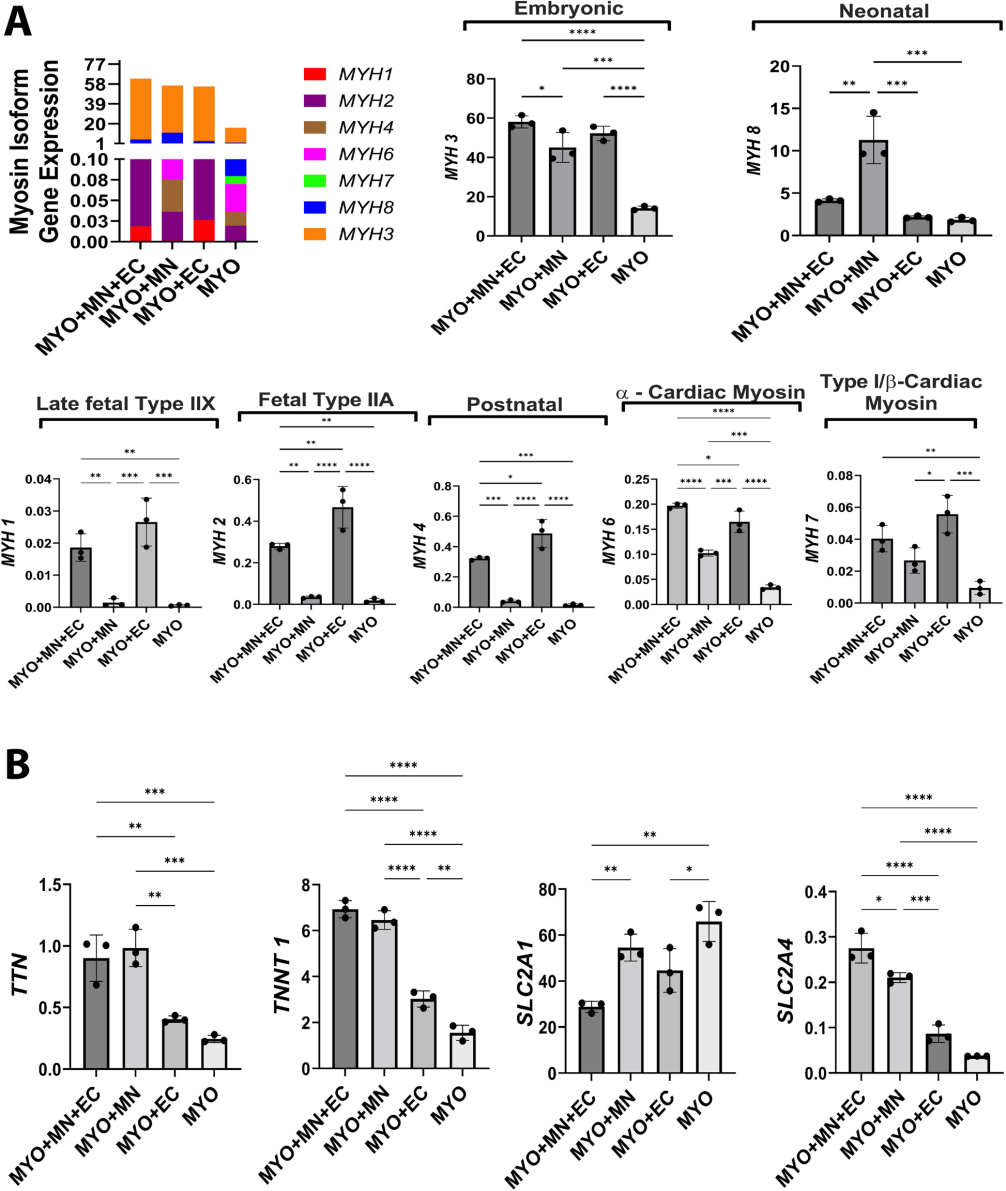
Genetic implications of myoneurovascular triculture. **A** qRT-PCR analysis of gene expression related to myosin heavy chain isoforms was performed on various culture combinations after 21 days. All the *MYH* gene isoforms analyzed are represented as colored grouped stack showing the predominance of *MYH3* (orange) and *MYH 8* (blue), whereas other isoforms were expressed at significantly lower levels. The presence of endothelial cells and motor neurons increased expression of embryonic *MYH3*, whereas motor neurons alone significantly upregulated expression of neonatal *MYH8* gene. Endothelial cells independently (MYO + EC) and in combination with motor neurons (MYO + MN + EC) lead to upregulation genes related to fast type II muscle fibers (*MYH1*, *MYH2*, and *MYH4*). **B** Innervation alone (i.e., MYO + MN) was found to upregulate genes pertaining to myocyte structural protein titin (*TTN*) as well as slow-type skeletal muscle-specific troponin T1 (*TNNT1*), and addition of endothelial cells on top of neuromuscular cocultures (MYO + MN + EC) did not significantly alter the expression of these genes. Further, endothelial cells downregulated expression of type 1 glucose transporter as evidenced by lower *SLC2A1* gene expression in MYO + EC and MYO + MN + EC groups. Interestingly, motor neurons and endothelial cells had an additive effect in upregulation of insulin-specific glucose transporter gene (*SLC2A4*) with MYO + MN + EC group exhibiting highest expression. Ordinary one-way ANOVA analysis with Tukey’s multiple comparison test was performed with *n* = 3 replicates per group, and *p* < 0.05 was considered for statistical significance

To further evaluate the functional significance of these tricultures, the expression pattern of genes related to type, structure, and glucose transporters of myofibers was studied. We found that across all experimental groups, embryonic *MYH3* and neonatal *MYH8* were the predominantly expressed myosin isoform gene with *MYH8* significantly upregulated in the presence of motor neurons in MN-MYO group (Fig. 5A). Endothelial cells, independent of motor neurons, promoted expression of various type II myofiber-specific genes like *MYH1*, *MYH2*, and *MYH4*, as well as *MYH7* which is specific for cardiac ventricles or type I-specific myofibers (Fig. 5A). Interestingly, motor neurons when cultured without endothelial cells had an inhibitory effect on the expression of all these genes. A more additive effect of motor neurons and endothelial cells were observed with regard to enhanced of expression alpha cardiac myosin gene — *MYH6* (Fig. 5A), gene encoding for contractile myofilament protein titin — *TTN* [20], and slow-type myofiber-specific troponin T1 gene — *TNNT1* [21, 22] in MYO + MN and MYO + MN + EC groups (Fig. 5B). Separately, genes related to glucose transporters (*SLC2A1* and *SLC2A4*) were also analyzed to understand neurovascular implications towards sensitivity to insulin in human iPSC-derived myocytes. *SLC2A1* gene expression was unaltered in the presence of motor neurons but significantly reduced by endothelial cells in MYO + EC and MYO + MN + EC groups (Fig. 5B). Interestingly, although overall *SLC2A4* expression was low, we did observe that the myoneurovascular triculture group (MYO + MN + EC) exhibited significantly higher level of insulin-regulated glucose transporter gene than other groups. These results highlight how coculture with neurovascular cells can lead to additive increase in expression of genes related to slow as well as fast-type myofibers with concomitant increase in insulin-sensitive *SCL2A4* gene in the triculture group (MYO + MN + EC (Fig. 5).

### Motor neurons and endothelial cells in combination promote expression of neuromuscular junction-related genes

**Fig. 6:**
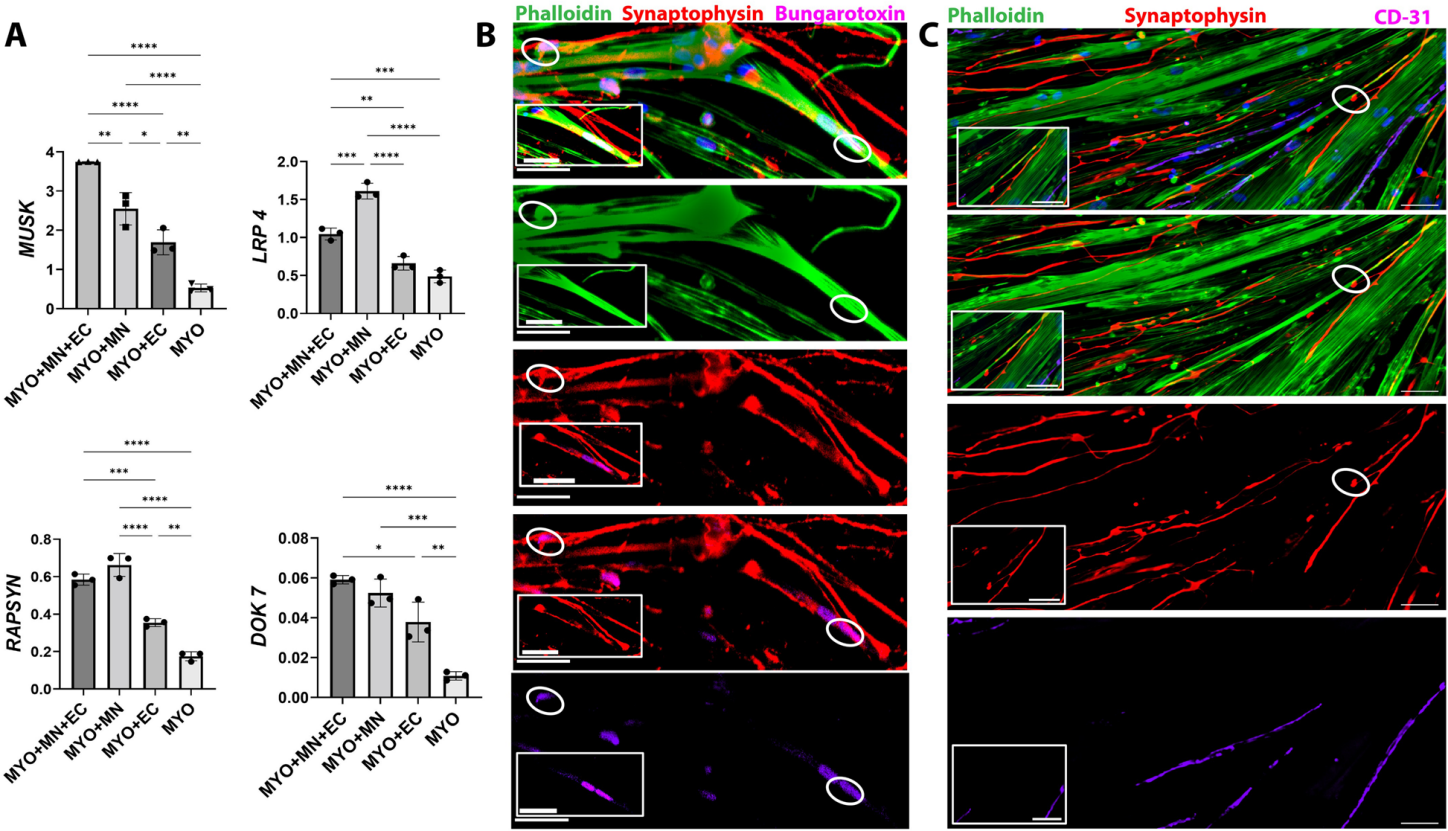
Expression of genes specific to neuromuscular junction and innervation of iPSC-derived skeletal myocytes. **A** Expression of genes related to different components of the neuromuscular apparatus was analyzed to understand effects of neural and endothelial cells on neuromuscular junction proteins. The presence of motor neurons by itself was sufficient to significantly increase expression of neuromuscular genes like *LRP4*, *RAPSYN*, and *DOK7*. However, addition of endothelial cells to neuromuscular cocultures downregulated *LRP4* while promoting expression of muscle-specific kinase (*MUSK*) gene. Ordinary one-way ANOVA analysis with Tukey’s multiple comparison test was performed with *n* = 3 replicates per group, and *p* < 0.05 was considered for statistical significance. **B–C** Human iPSC-derived motor neurons were observed to innervate skeletal myocytes in the **B** MYO + MN and **C** MYO + MN + EC groups. **B** In the MYO + MN group, points of innervation were identified upon colabelling for presynaptic marker, synaptophysin (red), and acetylcholine receptor-specific bungarotoxin (purple) indicating putative NMJs. **C** Similarly, in the triculture group (MYO + MN + EC), we found axons running parallel to and innervating myofibers. Points of contact between axon terminal and myofiber is demarcated by white ovals. Rectangular call out boxes denote zoom-in images near points of innervation. Scale bar — 50 μm

The neuromuscular junction (NMJ) is a complex structure comprising several key presynaptic and postsynaptic proteins. To better understand the effect of motor neurons and endothelial cells towards forming a stable neuromuscular apparatus, we analyzed the expression of genes encoding synaptic proteins like MUSK, LRP-4, DOK-7, and RAPSYN to understand the effect of motor neurons and endothelial cells towards forming a stable neuromuscular apparatus. Motor neurons and endothelial cells in combination (MYO + MN + EC) increased expression of *MUSK*, *LRP-4*, *DOK-7*, and *RAPSYN* (Fig. 6A). However, the presence of endothelial cells alone with myocytes was found to significantly inhibit expression of *LRP-4* in MYO + EC as compared to the innervated MYO + MN group (Fig. 6A). Motor neurons were found to innervate myofibers forming putative NMJ-like connections (Fig. 6B). We also observed spontaneously twitching myofibers in the presence of motor neurons (Supplementary Videos 1, 2), although no twitching was observed in myofibers cultured in the absence of neurons, further demonstrating the necessity of motor neurons in these conditions. These results establish that the combined presence of motor neurons and endothelial cells supports expression of key neuromuscular components.

### Juxtracrine signaling between myocytes — motor neurons — endothelial cells influence myocyte maturation.

**Fig. 7:**
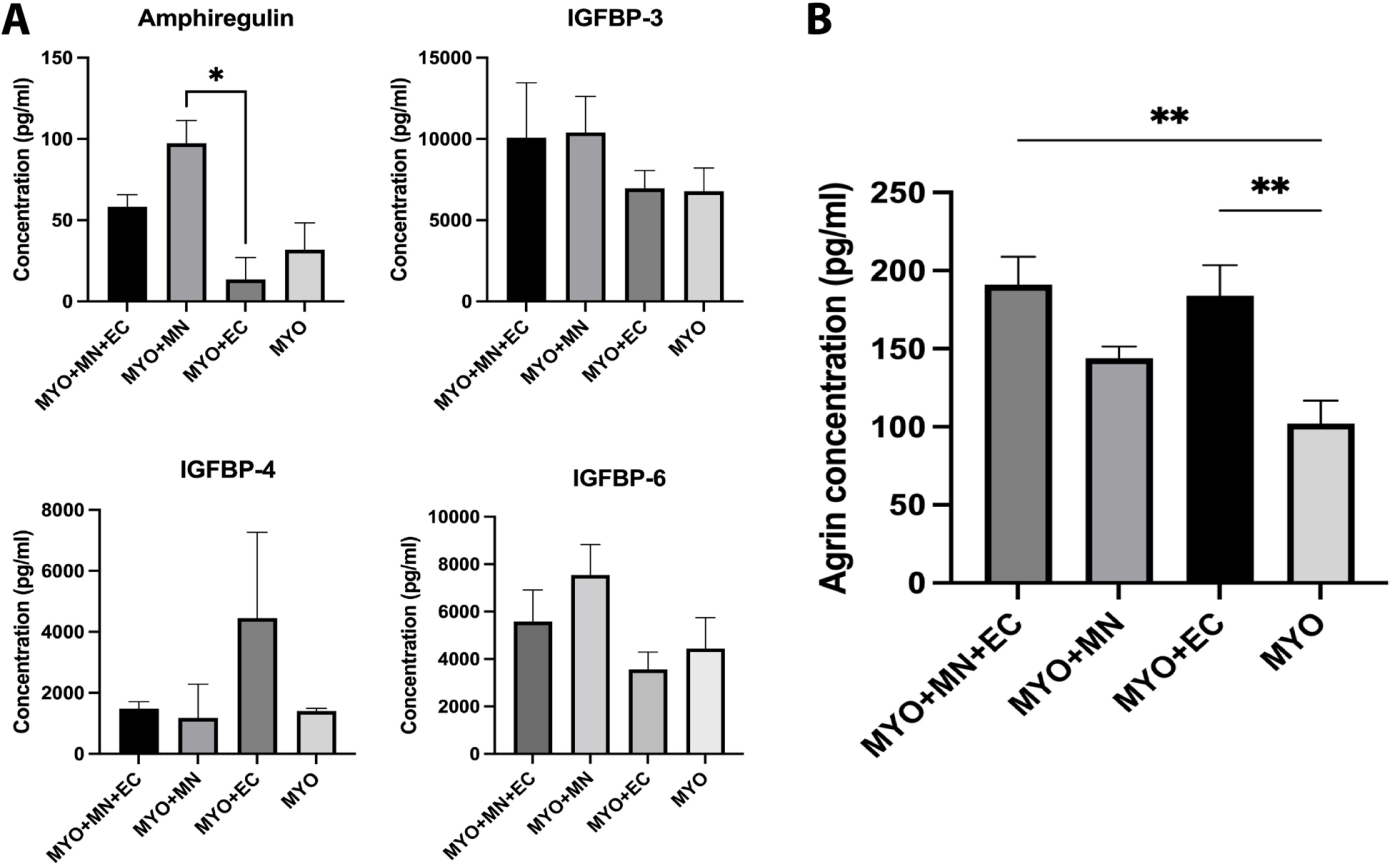
Growth factor expression pattern in myoneurovascular triculture. **A** Cell culture supernatant from the various groups was taken after 21 DIV and monitored for the secretion of growth factors like amphiregulin, insulin growth factor-binding proteins (IGFBP) 3, 4, and 6 subtypes. Motor neuron-myocyte coculture significantly increased production of amphiregulin, which is a potent mitogen of neural stem cells and has been shown to promote neurite outgrowth. IGFBPs regulate the activity of insulin-like growth factors (IGFs), wherein IGBP-4 is specifically inhibitory to IGF-1 and IGF-2. We observed high levels of extracellular IGFBP-4 when endothelial cells were cocultured with myocytes (MYO + EC) compared to other groups indicating that such a system can result in inhibiting IGF-1 and IGF-2-mediated effects on cells. Although not significant, the presence of motor neurons was found to increase levels of IGFBP-6, which is a regulator of IGF-2 signaling pathway. IGFBP-6 is significantly upregulated in spinal motor neurons post injury and has been found to be neuroprotective. We did not observe any significant difference in IGFBP-3 expression among the different experimental groups. Kruskal–Wallis test followed by Dunn's multiple comparison test was performed, *n* = 3/group was analyzed, and *p* < 0.05 was considered for significance. **B** Agrin expression by different cell combinations after 21 DIV was quantified from cell culture supernatants using an Agrin-specific ELISA assay. Endothelial cells alone (MYO + EC) as well as in combination with motor neurons (MYO + MN + EC) lead to elevated levels of Agrin expression as compared to myocyte monocultures. Ordinary one-way ANOVA with Tukey’s multiple comparison test was performed. *n* = 6/group was analyzed, and *p* < 0.05 was considered for significance

To define the molecular signatures influencing myocyte maturation, we collected cell culture supernatant at the terminal time point of 21 DIV from all experimental groups, and the levels of various secreted growth factors were evaluated by a high-throughput quantibody-based assay. Only those growth factors with levels in experimental groups higher than the control samples (base triculture media) were analyzed and reported. Motor neuron-myocyte coculture produced significantly high levels of amphiregulin, which is an epidermal growth factor receptor ligand [23] (Fig. 7A). Further, neuromuscular cocultures (MN-MYO) displayed elevated levels of insulin-like growth factor-binding proteins, IGFBPs (like IGFBP-3, IGFBP-6), which play a critical role in modulating the activity of insulin-like growth factors (IGF) (Fig. 7A). Although there were no statistically significant differences in IGFBP expression among the groups, we did notice a strong trend towards high IGFBP-4 secretion in myocyte-endothelial cocultures (MYO + EC) (Fig. 7A). Separately, levels of total Agrin secreted by the cells were measured by Agrin-specific ELISA assay on cell culture supernatant at 21 DIV. Endothelial cells significantly increased Agrin production in cocultures (MYO + EC) and tricultures (MYO + MN + EC) as compared to myocyte monoculture (MYO) (Fig. 7B).

## Discussion

The morphological, genetic, and molecular analyses in the present study strongly indicate that motor neurons and endothelial cells in combination promote formation of long multinucleated myotubes (Fig. 2, Fig. 3) exhibiting strong expression of neuromuscular junction-specific genes (*RAPSYN*, *DOK-7*, *LRP-4*) (Fig. 6A) along with putative neuromuscular junction points leading to spontaneously contractile myofibers (Fig. 6B, Supplementary Videos 1, 2). Endothelial cells alone, in the absence of neurons, were not supportive of fusion and led to stunted growth of myofibers as well as downregulated key Agrin receptor — *LRP-4*. These findings suggest that in the triculture system we describe, endothelial cells when cultured together with spinal motor neurons rather than in isolation promote myocyte maturation and expression of NMJ-related genes. The triculture system reported here can potentially be used as a test bed for further studies on human myoneurovascular development as well as for engineering composite soft tissue for musculoskeletal repair.

Neural and vascular implications on myocyte morphological parameters like fiber length, diameter, and fusion index have only previously been studied in isolation. Neuromuscular cultures have been used to study and model NMJs, neurodegenerative diseases, and control of bioactuators [12, 14, 16, 24–26]. It is also well established that the presence of motor neurons promotes formation of polynucleated myofibers [11, 27, 28]. Separately, endothelial cells have been shown to stimulate myogenesis in vitro by enhancing migration and proliferation of muscle progenitor cells as well as increase expression of myogenin indicating terminal differentiation [3]. However, endothelial cells did not enhance fusion of myocytes in 2D cocultures but considerably increased fusion index in 3D myovascular cocultures [3]. Coculture of endothelial cells and skeletal myocytes on scaffolds has been shown to form a tubular network of vessels that promote in vivo vascular integration of tissue-engineered muscles [29, 30]. It is noteworthy that human iPSC-derived endothelial cells have been found to exhibit a fivefold reduction in capillary network formation compared to HUVEC in vitro and a threefold reduction in the number of perfused vessels in vivo compared to HUVEC. Hence, in the present study, we opted for the established cell line — HUVEC as our endothelial cell source [31, 32]. Further, coculture of neural and endothelial cells has been used to model the neurovascular unit to better understand blood–brain-barrier functions [33]. The current study utilized tissue culture-coated two-dimensional (2D) surfaces to optimize and maintain a human myoneurovascular triculture as well as understand how neural and vascular input can influence muscle maturation independently and in combination.

There are limited reports attempting to understand the combined effect of neurovascular cells on muscle biology, disease, and regeneration. Maffioletti et al. and Pinton et al. in subsequent studies reported a protocol to generate multilineage skeletal muscles by differentiating iPSC stem cells into myogenic, neural, and vascular progenitor cells followed by coculture on 3D hydrogels to generate myofiber scaffold with neural and vascular networks [34, 35]. Through extensive characterization, they further showcased the importance of recreating the multicellular niche of skeletal muscle towards modelling muscular dystrophies and the potential application of such a platform in developing neuromuscular or musculoskeletal therapies. However, both the studies focused on developing a multilineage culture protocol for disease modelling but did not investigate the effect of these multiple cell types on each other. In contrast, the present study describes a myoneurovascular triculture system comprising human iPSC-derived skeletal myocytes (MYO) and motor neurons (MN) with primary human endothelial cells (EC) with the intention of investigating how motor neurons and endothelial cells independently as well as in combination affect myocyte development and maturation.

Directed non-transgenic differentiation of human pluripotent stem cells to skeletal myocytes usually produces spontaneously contracting myocytes that have reduced fusion efficiency and limited engraftment potential in vivo as compared to late fetal or adult stage human myocytes [18, 36–38]. Indeed, extensive comparative analysis reveals that such myocytes are like human fetal week 9 myocytes in functionality [18]. In this study, we generated SMPCs from an iPSC cell line following a previously published protocol that has been shown to produce myocytes resembling late-stage fetal myofibers and amenable to cryopreservation by 30 days [19] (Fig. 1). This differentiation protocol was subsequently combined with sorting for ERBB3^+^/NGFR^+^ to enrich for myogenic population as described by Hicks et al. [18]. We would like to point out that by day 4 of differentiation of SMPCs, we observed an increase in Pax-7 (Fig. 1B) along with robust expression of MYHC as evidenced by Fig. 1C indicating the presence of myofibers. The data indicates that we had a heterogenous pool of cells comprising fused myofibers as well as some unfused progenitor cells expressing Pax-7. The resulting SMPCs were cultured with motor neurons and endothelial cells independently or as a triculture to explore the effects of neural and endothelial cells on skeletal myocyte maturation. We designed the culture protocol to allow differentiation of myotubes prior to addition of other cells and formulated a triculture media such that myocyte survival and health were not compromised (Fig. 2A).

Morphological characterization of our cultures reveals that endothelial cells in combination with neurons (MYO + MN + EC) significantly increased myofiber fusion (MYO + MN) but were comparatively ineffective when cultured alone with myocytes (MYO + EC) (Fig. 2B, C). Neural stem cells have been found to increase the diameter and length of muscle progenitor cells when grown in coculture [28]. In the culture conditions used in our study, motor neurons and endothelial cells were observed to have an additive effect in increasing myofiber length in MYO + MN + EC triculture, whereas no significant change in myofiber diameter was observed (Fig. 2B, C). Further, motor neurons significantly promoted formation of anisotropic myofibers (Fig. 3). Previous work using 3D myovascular coculture demonstrated that myogenesis and angiogenesis are coupled via orchestrated production of apelin, oncostatin, and periostin [3]. Innervation has also been shown to induce muscle hypertrophy most probably by activating Ras-dependent pathways [39]. Although we did not directly investigate these pathways, we speculate that these may be possible mechanisms by which neurons and endothelial cells in our triculture system may be influencing muscle fiber length and fusion.

In our attempt to optimize cell culture media and cell plating sequence, we noted that although motor neurons and endothelial cells could be cultured separately in their prescribed commercial media, our triculture media recipe was not conducive for such monocultures. We found that conditioning our triculture media with myocytes augmented survival and health of motor neurons and endothelial cells (Fig. 4). Furthermore, conditioning the triculture media with neuromuscular coculture did not have any adverse effect on endothelial cell growth (Fig. 4A). This prompted us to design a plating sequence where myocytes were added first and allowed to differentiate before adding motor neurons and subsequently endothelial cells (Fig. 2A). The beneficial role of skeletal myocyte conditioning towards survival and growth of neurovascular cells can be explained by how muscle-derived exosomes have been shown to enhance endothelial cell function via reactive oxygen species-activated nuclear factor-κB signaling [40] as well as improve motor neuron regeneration [41].

Skeletal muscle fibers are comprised of the myofibrillar proteins actin and myosin and regulatory proteins like troponin, tropomyosin, and contractile proteins like titin [20, 42]. Different isoforms of these proteins determine the formation of slow or fast myofibers. Slow or type I myofibers are oxidative (i.e., use aerobic respiration), produce lower contractile force, but have higher endurance and resistance to fatigue. Fast twitch or type II myofibers can either be oxidative or glycolytic (i.e., use anerobic glycolysis) that generate more contractile force but get fatigued sooner. In the present study, we investigated differential expression of several myosin isoforms (*MYH1,2,3,4,8*), slow-type troponin T1 (*TNNT1*), titin (*TTN*), alpha-cardiac myosin (*MYH6*), beta-cardiac myosin, or type I-specific myosin (*MYH7*) to understand neurovascular implications on skeletal myofiber properties. Endothelial cells were found to promote expression of type II-specific myosin (*MYH1, 2, and 4*) as well as type I-specific *MYH7*, whereas motor neurons seemed to reduce expression of *MYH1*, *MYH2*, and *MYH4* and upregulate *MYH8* (Fig. 5A). We further observed that our culture conditions (media, density, and duration) resulted in predominant expression (approximately 50–100-fold more than any other gene) of immature *MYH3* and *MYH8*. The expression pattern indicates that motor neurons push the myocytes towards a more embryonic and neonatal phenotype. Although the exact reason behind such a genetic expression pattern is unclear, we speculate that our time point of analysis (21 days) was too acute to allow expression of more matured MYH isoforms like *MYH1*, *2*, and *4*.

Troponin T1 encoded by the *TNNT1* gene is expressed exclusively in type I slow muscle fibers [21, 43]. Troponin and titin play a pivotal role in excitation–contraction-relaxation of both cardiac and skeletal muscles [20, 21, 44, 45]. We observed that motor neurons and endothelial cells in combination increased the expression of both titin and slow-type troponin T1 genes in myoneurovascular tricultures indicating a propensity towards generation of slow-type I myofibers (Fig. 5B). Interestingly, both endothelial cells and motor neurons significantly increased expression of *MYH6* gene in the MYO + MN, MYO + EC, and MYO + MN + EC groups (Fig. 5B). Although *MYH6* gene encodes for alpha-cardiac myosin, it is also present in certain specialized craniofacial skeletal muscles like the masseter muscle in humans which predominantly comprise slow-type I myofibers [46–48]. However, considering we observe high expression of skeletal muscle-specific *TNNT1* and various other NMJ apparatus-related genes (Fig. 5B), we conclude that the myocytes resemble a more craniofacial skeletal muscle subtype and not cardiac. Our data indicates that iPSC motor neurons inhibit type II- and type I-specific myosins (*MYH1, 2, 4, and 7*), promote slow-type troponin T1, and push skeletal myocytes towards a more neonatal phenotype (*MYH8*). This is in congruence with a previous report showing motor neurons increased *TNNT1* and *MYH8* in 3D neuromuscular cocultures [49]; however, the exact molecular mechanisms behind such an effect are unclear.

We further studied the expression of glucose transporter genes and notably found that motor neurons and endothelial cells in combination upregulated expression of insulin-sensitive *SLC2A4* gene (Fig. 5B). *SLC2A4* encodes for glucose transporter 4 (GLUT-4) which is primarily expressed in striated muscle and is responsible for insulin-regulated uptake of glucose by cells. Although the role of endothelial cell *SLC2A4* expression is not well characterized, there is evidence that regenerating motor neurons in the facial nerve upregulates *SLC2A4* expression through IGF-1-induced Akt phosphorylation [50] (Fig. 5A).

The NMJ is a highly specialized structure between a motor nerve terminal and a skeletal muscle fiber that is responsible for signal transduction and muscle contraction. It comprises several key molecules like Agrin, LRP, MuSK, Rapsyn, Dok-7, and others that span across the presynaptic terminal, synaptic cleft, and acetylcholine receptors (AChRs) in the postsynaptic muscle membrane [51]. Neuronal isoform of Agrin is released from the presynaptic terminal into the basal lamina where it binds to LRP-4 which in turn activates muscle-specific kinase (MuSK). This triggers a cascade of intracellular pathways involving key adapter protein Dok-7 that leads to clustering of AChRs by scaffolding protein Rapsyn [51, 52]. As expected, we found that motor neurons independently as well as in combination with endothelial cells upregulated expression of NMJ-specific genes — *Dok-7*, *MuSK*, *LRP-4*, and *RAPSYN* (Fig. 6A). Interestingly, we found that the myocyte coculture with only endothelial cells significantly downregulated Agrin receptor, *LRP-4* (Fig. 6A). As the effect of endothelial cells on NMJ-related genes has yet to be studied extensively, the exact mechanism underlying the impact of endothelial cells on *LRP-4* gene expression is not clear.

When NMJs were labeled with fluorogenic α-bungarotoxin, a specific marker of nicotinic acetylcholine receptors (nAChR) known to be densely expressed in the NMJ postsynaptic membrane, bright fluorescence was observed at end plates and colocalized with synaptophysin immunoreactivity, which is a presynaptic marker (Fig. 6B and C). These results led us to hypothesize that both the neuronal cells and the postsynaptic membranes present in myotubes of our human coculture model are forming “putative NMJs”.

Critical methodology to demonstrate the emergence of functional NMJs would involve functional blocking experiments, for instance using curare. These experiments should be included in future characterization of these co- and tri-culture systems, most likely at later timepoints to allow for functional maturity of the myofibers and NMJs. Because these experiments have yet to be performed, note that we currently refer to axonal-bungarotoxin-positive regions as “putative NMJs” or “points of innervation” rather than as “functional NMJs.”

We further investigated the production of multiple key biomolecules like amphiregulin, insulin-like growth factor-binding proteins (IGFBP-3, IGFBP-4, IGFBP-6), and Agrin across the various groups to understand the juxtracrine signaling between myocytes, motor neurons, and endothelial cells. Amphiregulin is a ligand epidermal growth factor receptor (EGFR) that is critical for EGFR, ERBB3, and Akt signaling pathways [23]. Interestingly, amphiregulin has been shown to enhance in vitro and in vivo cellular reprogramming by accelerating proliferation and the mesenchymal-epithelial transition via EGFR signaling [53]. As our iPSC-derived myocytes were enriched for ERBB3 + cells, it is interesting to note that coculture of the myocytes with motor neurons significantly enhanced production of amphiregulin (Fig. 7A). Amphiregulin has also been found to be a potent mitogen for neural stem cells [54] while promoting neuronal survival and potentiating muscle repair by facilitating myogenesis and differentiation [55, 56]. On the other hand, insulin-like growth factors IGF-1 and IGF-2 are crucial for skeletal muscle differentiation [57–61], regulating sprouting of endothelial cells [62], and promoting motor neuron survival [63, 64]. IGFs are secreted by a variety of cells including skeletal muscle and vascular cells, although motor neurons specifically have not been shown to produce IGF. A variety of IGF-binding proteins (IGFBPs) modulate activity of IGFs, wherein IGFBP-4 is a known inhibitor of both IGF-1 and IGF-2 signaling [62, 65]. Further, IGFBPs secreted by skeletal muscles inhibit IGF-induced stimulation of protein [66] and DNA synthesis [67]. Molecular analysis of our cultures shows that endothelial cells drive-up production of IGFBP-4 in MYO + EC cocultures (Fig. 7A) indicating that such a system can result in inhibition of IGF-1- and IGF-2-mediated effects on cells. Although not significant, we noted a trend in neuromuscular cocultures (MYO + MN) towards increased production of IGFBP-6, which regulates IGF-2 signaling (Fig. 7A) [68]. In a separate study on skeletal muscle differentiation from mesenchymal stem cells, IGFBP-6 was found to increase expression of pluripotency markers as well as muscle differentiation markers at early stages of differentiation even in the absence of IGF-2 [59, 69]. We also monitored extracellular production of Agrin in our cell culture samples through ELISA-based assay. Agrin is a key mediator of acetylcholine receptor clustering secreted by innervating motor neurons and plays a critical role in formation and maintenance of neuromuscular junctions [70]. We expected cultures with motor neurons to have high levels of Agrin which would concur with our data for expression of genes related to neuromuscular junction. On the contrary, we found that endothelial cells significantly increased Agrin production in MYO + EC as well as MYO + MN + EC groups (Fig. 7B). It is noteworthy that different isoforms of Agrin are secreted across a variety of organs particularly in the brain, lungs, and muscle [71]. Specifically, both microvascular and macrovascular cells including HUVEC secrete endothelial Agrin that has been shown to promote angiogenesis by stabilizing VEGF-R2 through the LRP-4-MuSK pathway [72]. Different isoforms of alternatively spliced Agrin are produced by muscles and nerves. Even within the neuronal isoforms, the one containing Z exons (Z +) is critical for postsynaptic differentiation, whereas other neural isoforms containing heparin-binding site (Y +) and muscle-derived isoforms are not effective in synaptogenesis [73, 74]. High expression of Agrin in MYO + EC and MYO + MN + EC groups thus indicates the predominant production of endothelial- and muscle-derived Agrin (Fig. 7B). Although in vitro models of NMJs comprising iPSC-derived motor neurons and myocytes have been shown to be responsive to exogenous Agrin treatment [75], we did not find any report showing secretion of Agrin from iPSC-derived motor neurons. It is possible that iPSC-derived motor neurons are usually not mature enough to secrete the appropriate isoform of neural Agrin. Further, our ELISA-based assay was not sensitive to specific Agrin isoform and could only measure total Agrin present.

Our extensive morphological, genetic, and molecular analyses suggest that innervation of skeletal myocytes through motor neurons in combination with endothelial cells can significantly increase expression of slow-type troponin T1 (*TNNT1*), contractile myofilament (*TTN*), insulin-sensitive transporter *SLC2A4*, and multiple neuromuscular junction genes like *MUSK*, *LRP-4*, *Dok-7*, and *RAPSYN*, ultimately forming longer spontaneously contractile myofibers with higher fusion. Innervation was observed to have such beneficial effects towards muscle maturation despite motor neurons not producing significant amounts of Agrin. This is probably due to the enhanced expression of Dok-7 gene which has been shown to be able to activate MuSK and clustering of AChRs independent of Agrin [52]. On the other hand, endothelial cells, although significantly improving Agrin production, did not ultimately lead to genetic or morphological alterations that would indicate maturation of myofibers or NMJ apparatus. We speculate that low expression of critical Agrin receptor gene, *LRP-4*, and high levels of IGF signaling inhibitor, IGFBP-4, in myovascular cultures contributed towards stunted myofibers and restricted fusion.

Although the triculture system described in this study showcases morphological, genetic, and molecular effects of neurovascular cells on skeletal myocytes, such findings are restricted to the cell culture protocol followed here. Hence, any changes to cell culture parameters like cell source, experimental duration, cell seeding ratio and sequence, or media composition may lead to different findings. Evaluating the effects of all these numerous variables on multiple cell types is not trivial. Hence, our experimental focus was on developing a triculture setup that ensures highest myocyte viability and limited to investigating the effects of motor neurons and endothelial cells on myocytes.

## Conclusions

This study describes a novel myoneurovascular platform comprising a triculture of human iPSC-derived myocytes and motor neurons with primary human endothelial cells mimicking the multicellular niche of skeletal muscle. We describe a triculture media comprising equal portions of muscle differentiation media, motor neuron media, and vascular media as a suitable media for culturing all three cell types and as evidenced through phenotype-specific expression of markers without compromising myocyte growth. Through morphological-, genetic-, and molecular-level experiments, we demonstrate that combined presence of neural and vascular cells facilitates robust myofiber fusion and neuromuscular gene expression. We also show for the first time that a triculture setup comprising skeletal myocytes, endothelial cells, and motor neurons can significantly increase expression of both a key neuromuscular gene (*MuSK*) and the insulin-sensitive glucose transporter gene (*SLC2A4*), as well as lead to longer, aligned polynucleated myofibers. The triculture paradigm described here may be used to develop a 3D myoneurovascular platform to study disease and physiology related to neuromuscular and myovascular systems as well as engineer composite soft tissue for repair of severe musculoskeletal trauma.

### Supplementary Information

**Additional file 1: Supplementary Video 1.** Spontaneous twitching of myofibers observed at 21 DIV in MYO + MN group

**Additional file 2: Supplementary Video 2.** Spontaneous twitching of myofibers observed at 21 DIV in MYO + MN + EC group.

## Data Availability

Data supporting the conclusions of this paper are available from the corresponding author upon reasonable request.
